# Variability of the perception of dyspnea in healthy subjects assessed through inspiratory resistive loading*

**DOI:** 10.1590/S1806-37132015000004409

**Published:** 2015

**Authors:** Bruna Ziegler, Andréia Kist Fernandes, Paulo Roberto Stefani Sanches, Glauco Luís Konzen, Paulo de Tarso Roth Dalcin

**Affiliations:** Universidade Federal do Rio Grande do Sul, Porto Alegre Hospital de Clínicas, Porto Alegre, Brazil. Porto Alegre Hospital de Clínicas, Universidade Federal do Rio Grande do Sul - UFRGS, Federal University of Rio Grande do Sul - Porto Alegre, Brazil; Universidade Federal do Rio Grande do Sul, Porto Alegre Hospital de Clínicas, Porto Alegre, Brazil. Porto Alegre Hospital de Clínicas, Universidade Federal do Rio Grande do Sul - UFRGS, Federal University of Rio Grande do Sul - Porto Alegre, Brazil; Universidade Federal do Rio Grande do Sul, Porto Alegre Hospital de Clínicas, Porto Alegre, Brazil. Porto Alegre Hospital de Clínicas, Universidade Federal do Rio Grande do Sul - UFRGS, Federal University of Rio Grande do Sul - Porto Alegre, Brazil; Universidade Federal do Rio Grande do Sul, Porto Alegre Hospital de Clínicas, Porto Alegre, Brazil. Porto Alegre Hospital de Clínicas, Universidade Federal do Rio Grande do Sul - UFRGS, Federal University of Rio Grande do Sul - Porto Alegre, Brazil; Universidade Federal do Rio Grande do Sul, School of Medicine, Porto Alegre, Brazil. Universidade Federal do Rio Grande do Sul - UFRGS, Federal University of Rio Grande do Sul - School of Medicine, Porto Alegre, Brazil

**Keywords:** Dyspnea, Respiratory function tests, Exercise test

## Abstract

**Objective:**

Few studies have evaluated the variability of the perception of dyspnea in healthy subjects. The objective of this study was to evaluate the variability of the perception of dyspnea in healthy subjects during breathing against increasing inspiratory resistive loads, as well as to assess the association between the level of perception of dyspnea and the level of physical activity.

**Methods:**

This was a cross-sectional study involving healthy individuals 16 years of age or older. Subjects underwent inspiratory resistive loading testing, in which the level of perception of dyspnea was quantified with the modified Borg scale. We also determined body mass indices (BMIs), assessed maximal respiratory pressures, performed pulmonary function tests, applied the international physical activity questionnaire (IPAQ)-long form, and conducted six-minute walk tests (6MWTs). The level of perception of dyspnea was classified as low (Borg score < 2), intermediate (Borg score, 2-5), or high (Borg score > 5).

**Results:**

We included 48 healthy subjects in the study. Forty-two subjects completed the test up to a load of 46.7 cmH_2_O/L/s. The level of perception of dyspnea was classified as low, intermediate, and high in 13, 19, and 10 subjects, respectively. The level of perception of dyspnea was not significantly associated with age, gender, BMI, IPAQ-long form score, maximal respiratory pressures, or pulmonary function test results.

**Conclusions:**

The scores for perceived dyspnea induced by inspiratory resistive loading in healthy subjects presented wide variability. The perception of dyspnea was classified as low in 31% of the subjects, intermediate in 45%, and high in 24%. There was no association between the level of perception of dyspnea and the level of physical activity (IPAQ or six-minute walk distance).

## Introduction

Breathlessness, or dyspnea, is the subjective experience of respiratory discomfort and consists of qualitatively distinct sensations that vary in intensity. This symptom has multidimensional aspects, involving physiological, psychological, social, and environmental factors that result in a behavioral response.^(^ 1 ^)^ In patients with pulmonary impairment, dyspnea is often accompanied by physical inactivity, decreased exercise capacity, and impaired quality of life.^(^ 2 ^-^ 5 ^)^

Dyspnea is a common problem that is seen in up to half of all acute cases admitted to tertiary care hospitals.^(^ 1 ^)^ Assessment of the multidimensional aspects of dyspnea has become more important in recent years. Dyspnea is an important warning symptom and is considered a predictor of hospitalization and mortality in patients with chronic lung disease, mainly in a subgroup of patients with a blunted perception of dyspnea.^(^ 6 ^,^ 7 ^)^

Healthy subjects can experience dyspnea in different situations-at high altitudes, after breath-holding, during stressful situations that cause anxiety or panic, and (most commonly) during strenuous exercise.^(^ 8 ^)^ Dyspnea occurs in a highly variable way in comparison with the levels of pathophysiology. However, little is known about the variability of the perception of dyspnea in healthy subjects.^(^ 7 ^)^

Various studies have used inspiratory resistive loading in order to evaluate the perception of dyspnea and to investigate factors associated with increased or decreased sensitivity to dyspnea. ^(^ 9 ^-^ 15 ^)^ Testing with inspiratory resistive loading involves the use of a circuit within which loads of increasing magnitudes can be created, thus inducing the sensation of dyspnea by increasing inspiratory effort and the overall work of breathing. Subjects quantify the severity of dyspnea using instruments such as the Borg scale.^(^ 11 ^,^ 12 ^,^ 14 ^)^

The objective of this study was to evaluate the variability of the perception of dyspnea in healthy subjects during inspiratory resistive loading. A secondary objective was to investigate the association between the perceived severity of dyspnea and the level of physical activity. 

## Methods

### Study design

We conducted a prospective cross-sectional study designed to evaluate the perception of dyspnea in healthy subjects. For each subject, over the course of a single day, we quantified the perceived severity of dyspnea during inspiratory resistive loading, determined maximal respiratory pressures, performed pulmonary function tests, and conducted a nutritional evaluation, as well as applying the long form of the International Physical Activity Questionnaire (IPAQ-long form) and the six-minute walk test (6MWT). The study was approved by the Research Ethics Committee of the *Hospital de Clínicas de Porto Alegre* (HCPA, Porto Alegre *Hospital de Clínicas*), in the city of Porto Alegre, Brazil (Protocol no. 08-063). All participants (or their parents or legal guardians) gave written informed consent. 

### Population

Using notices posted in the HCPA and online announcements, we recruited 48 healthy subjects. We excluded subjects who were < 16 years of age, as well as those who were pregnant, had acute conditions affecting the respiratory tract, were current or former smokers, or had any chronic medical condition, such as asthma, chronic pain, heart disease, musculoskeletal disorders, and traumatic injury. Otherwise, all subjects who volunteered during the period of the study were consecutively enrolled. 

### Measurements and procedures

Healthy subjects underwent perception of dyspnea testing involving inspiratory resistive loading.^(^ 16 ^)^ Before the tests, subjects were familiarized with the apparatus and measurement procedures. After receiving standardized instructions, subjects were seated in a comfortable chair and acclimatized to the setting. Wearing a nose clip, subjects breathed through a mouthpiece in a system composed of a two-way non-rebreathing valve (Hans Rudolph, Shawnee, KS, USA). A circular plastic mouthpiece (with eight different orifices) was employed in order to generate inspiratory resistive loads of increasing magnitude (0.6, 7.0, 15.0, 25.0, 46.7, 67.0, and 78.0 cmH_2_O/L/s, calculated according to a constant flow of 300 mL/s). The sensation of dyspnea was assessed during the inspiratory resistive loading. After breathing at each level of resistance for 2 min, the subjects were questioned about the feeling of shortness of breath (dyspnea), as quantified with the modified Borg scale,^(^ 17 ^)^ ranging from 0 (no dyspnea) to 10 (maximum severity of dyspnea). To monitor the effects of dyspnea induction, we monitored inspiratory pressure, inspiratory time and respiratory rate continuously at the mouthpiece using computer software developed by the HCPA Department of Engineering. Exhalation was not loaded. Subjects were free to choose their respiratory rate, volume, and flow, in order to have as natural a breathing pattern as possible.

The functional capacity of subjects was measured with the 6MWT, which was conducted in accordance with the guidelines established by the American Thoracic Society and the Brazilian Thoracic Association.^(^ 18 ^,^ 19 ^)^ Following a standardized protocol, subjects walked along a flat 30-m track established in a corridor. The subjects were instructed to walk as far as possible for 6 min under the supervision of a physiotherapist. The physiotherapist encouraged subjects with the standardized statements "you are doing well" or "keep up the good work", but was asked not to use other phrases. The total six-minute walk distance (6MWD) was recorded. The pre- and post-6MWT SpO_2_ were measured with a pulse oximeter (NPB-40; Nellcor Puritan Bennett, Pleasanton, CA, USA). We also recorded pre- and post-6MWT scores on the modified Borg scale.^(^ 17 ^)^

Pulmonary function tests were performed with a computerized spirometer (MasterScreen, v 4.31; Jaeger, Würtzburg, Germany). We recorded FVC, FEV_1_, and the FEV_1_/FVC ratio, in triplicate, and the best of the three was selected for analysis. All parameters are expressed as percentages of the values predicted for age, stature, and gender. ^(^ 20 ^)^ Nutritional status was classified on the basis of the body mass index (BMI), determined by dividing weight (in kg) by height (in m^2)^.

Maximal respiratory pressures were used as indexes of respiratory muscle strength. Pressure measurements were made in the seated position with a digital manometer (Microhard MVD300, version 1.0; Globalmed, Porto Alegre, Brazil). All subjects wore nose clips and were instructed to press their lips tightly against the mouthpiece to prevent air leakage during the pressure measurements.

We measured MIP at RV and MEP at TLC. The pressures measured were maintained for at least 1 s. Five determinations were made, with a suitable rest interval between each determination, until a plateau value had been reached and no further learning effect was seen. Once the operator was satisfied, the maximum values of two maneuvers that varied by less than 10% were recorded. The MIP and MEP were expressed in cmH_2_O and as percentages of the predicted values. We obtained the predicted values for adolescents and adults from Wilson et al. and Neder et al., respectively. ^(^ 21 ^,^ 22 ^)^ On the basis of the scores on the IPAQ-long form,^(^ 23 ^)^ the level of physical activity was categorized as low, moderate, or high.

### Statistical analysis

Data are expressed as number (percentage), mean ± standard deviation, or median (interquartile range). We divided the subjects into three groups, by the level of perception of dyspnea, according to the tertiles of Borg scores generated at an inspiratory resistive load of 46.7 cmH_2_O/L/s: low perception group (Borg score < 2; n = 13), intermediate perception group (Borg score 2-5; n = 19), and high perception group (Borg score > 5; n = 13). The inspiratory resistive load of 46.7 cmH_2_O/L/s was selected because it generated high dyspnea scores with little drop-off.

Categorical comparisons were performed with the chi-square test for proportions. Continuous variables with normal distribution were compared with one-way ANOVA for quantitative variables. Ordinal variables were compared with the Kruskal-Wallis H test. Kaplan-Meier curves were used in order to profile the subjects during the perception of dyspnea test at the different inspiratory resistive loads. To compare males and females in terms of the dyspnea scores during the inspiratory resistive loading, we used a generalized linear model. Correlations were determined using Spearman's rank correlation coefficient.

Data analysis was performed with the IBM SPSS Statistics software package, version 18.0 (IBM Corporation, Armonk, NY, USA). The level of statistical significance was set at p < 0.05. All statistical tests were two-tailed (α = 0.05 and 1−β = 90%).

## Results

From February 2010 to November 2012, we screened 54 subjects. We excluded 6 subjects: 2 presented abnormal spirometry values; 1 dropped out because of anxiety at the outset of the testing; and 3 failed to complete all required examinations. Therefore, 48 healthy subjects (19 males and 29 females) were included in the study. All of the subjects were White, the mean age was 31.2 ± 12.1 years (range, 16-61 years), and the mean BMI was 23.5 ± 3.4 kg/m^2^. Spirometry values (in % of predicted) were as follows: FEV_1_, 96 ± 12%; FVC, 95.1 ± 11.2%; and FEV_1_/FVC ratio, 100.6 ± 8.3%. The 6MWD was 579.2 ± 72 m.

**Figure 1:**
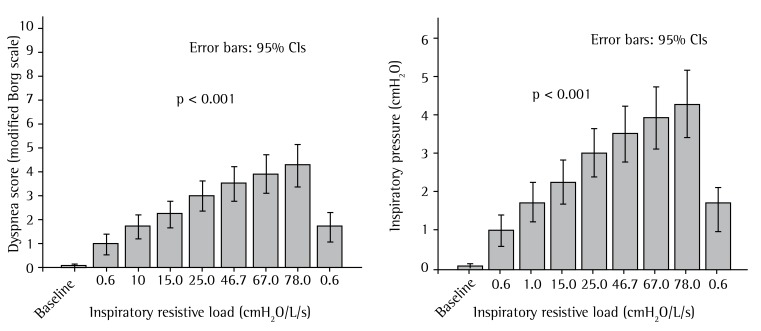
Dyspnea scores on the modified Borg scale, together with inspiratory pressures, at increasing inspiratory resistive loads in healthy subjects.

**Figure 2:**
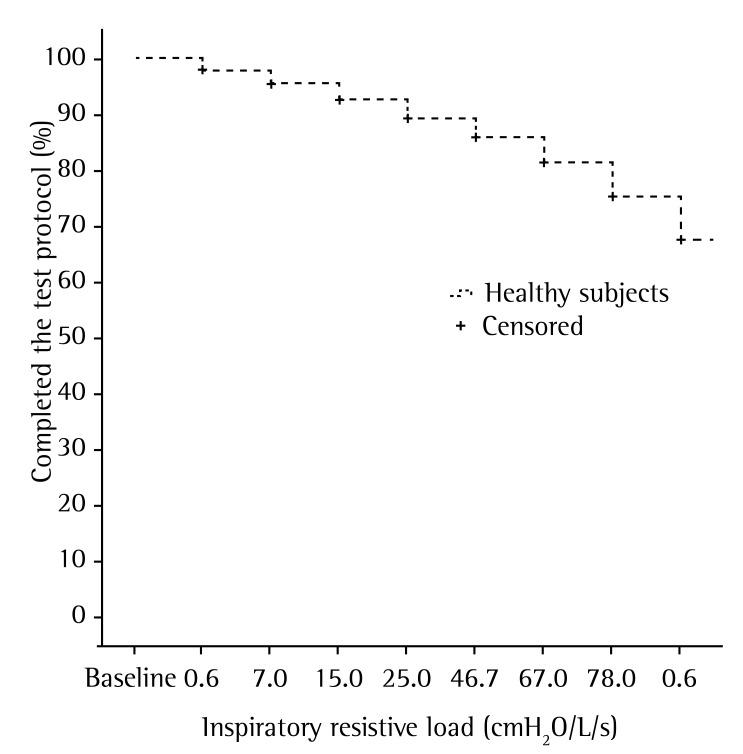
Kaplan-Meier analysis for interruption of the perception of dyspnea test at increasing inspiratory resistive loads in healthy subjects.

Figure 1 shows the modified Borg dyspnea score and inspiratory pressure at the various inspiratory resistive loads (p < 0.001). Figure 2 presents the Kaplan-Meier analysis of interruption of the perception of dyspnea test at increasing inspiratory resistive loads. Thirty-eight subjects (79.2%) completed the entire test (all inspiratory resistive loads), and 10 (20.8%) did not, because of the following symptoms: dyspnea (n = 3); respiratory fatigue (n = 3); headache (n = 2); drooling (n = 1); and dry throat (n = 1).

**Figure 3:**
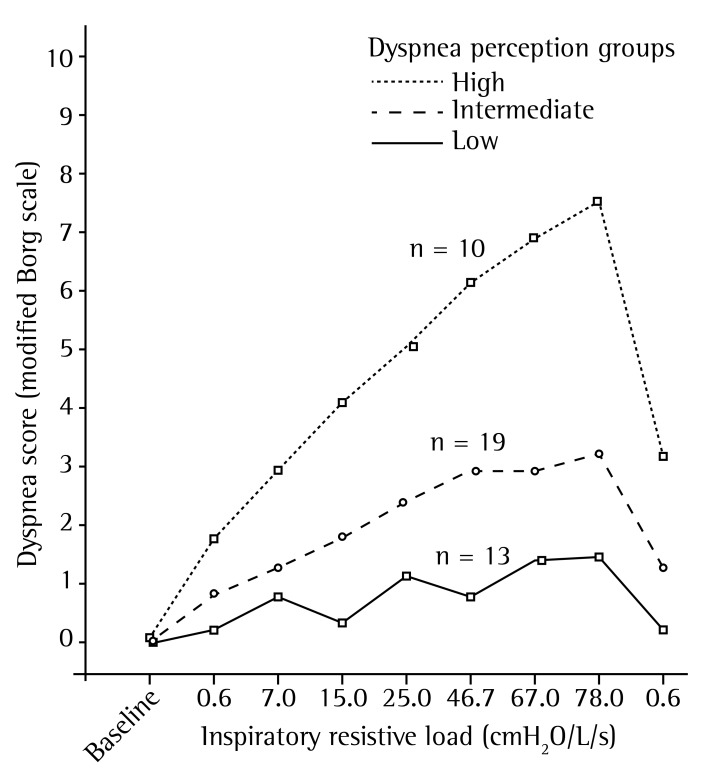
Patients stratified by the level of the perception of dyspnea (tertiles of scores on the modified Borg scale), with a focus on the differences at an inspiratory resistive load of 46.7 cmH2O/L/s. Dyspnea perception groups (modified Borg scale scores): low (< 2), intermediate (2-5), and high (> 5).

Figure 3 shows the perception of dyspnea groups, by tertiles of the scores on the modified Borg dyspnea scale scores generated at an inspiratory resistive load of 46.7 cmH_2_O/L/s. Forty-two subjects continued the test up to that load. The perception of dyspnea was classified as low (Borg score < 2), intermediate (Borg score, 2-5), and high (Borg score > 5) in 13, 19, and 10 subjects, respectively.

To compare males and females in terms of the dyspnea scores during the inspiratory resistive load testing, we used a generalized linear model. Although there was a statistically significant difference among the various inspiratory resistive loads (p < 0.001), there was no significant difference for gender (p = 0.590) or for the interaction between increasing inspiratory resistive loads and gender (p = 0.253).

Table 1 presents the characteristics of the subjects according to the level of perception of dyspnea. We found that the level of perception of dyspnea was not significantly associated with age, gender, BMI, IPAQ-long form score, maximal respiratory pressures, or pulmonary function test results. In addition, the inspiratory pressure at the various inspiratory resistive loads did not differ among the groups.

**Table 1:** Characteristics of healthy subjects, according to the level of perception of dyspnea.a

Variable	All	Perception of dyspnea			p^b^
		Low	Intermediate	High	
	(n = 42)	(n = 13)	(n = 19)	(n = 10)	
Age (years)	31.5 ± 11.5	34 ± 11.2	28.6 ± 11.3	33.8 ± 12.4	0.345
Gender (male/female), n/n	15/27	5/8	7/12	3/7	0.907
BMI (kg/m²)	23.3 ± 3.2	24.7 ± 2.8	22.7 ± 3.4	22.6 ± 2.8	0.150
Level of physical activity^c^					
Low, n (%)	7 (16.7)	3 (7.1)	3 (7.1)	1 (2.4)	
Moderate, n (%)	14 (33.3)	5 (11.9)	6 (14.3)	3 (7.1)	
High, n (%)	21 (50.0)	5 (11.9)	10 (23.8)	6 (14.3)	
MIP (cmH_2_O)	100.3 ± 36.6	105.2 ± 28.3	99.2 ± 39.4	95.1 ± 44.7	0.820
MEP (cmH_2_O)	109.8 ± 29.1	114.7 ± 18.4	111.8 ± 32.8	96.8 ± 33.4	0.364
PEF (% of predicted)	93.3 ± 15.4	97.5 ± 10.7	87.9 ± 18	97.9 ± 13.2	0.126
FEV_1_ (% of predicted)	96.2 ± 11.9	99.2 ± 12.5	94.6 ± 13.2	94.8 ± 8.4	0.560
FVC (% of predicted)	95.2 ± 10.6	98.2 ± 11	94.4 ± 11.2	92.6 ± 8.4	0.423
FEV_1_/FVC ratio (% of predicted)	101.1 ± 6.9	100.8 ± 5.7	100.8 ± 7.9	100.2 ± 6.8	0.859
Total 6MWD (m)	577 ± 70.9	548.6 ± 79.8	601 ± 67.2	568.3 ± 53.9	0.109
Pre-6MWT (at-rest) SpO_2_ (%)	98.2 ± 1.2	98.1 ± 1	98.3 ± 1.1	98 ± 1.2	0.753
Post-6MWT SpO_2_ (%)	97.7 ± 1.6	97.9 ± 2.0	97.9 ± 1.2	97.2 ± 1.6	0.504
Post-6MWT oxygen desaturation (%)	0.4 ± 1.8	0.2 ± 1.9	0.4 ± 1.5	0.8 ± 2.2	0.692
IP (cmH_2_O) at an IRL of 0.6 cmH_2_O/L/s	3.2 ± 2.4	3.4 ± 1.6	3.3 ± 3.1	2.9 ± 1.8	0.852
IP (cmH_2_O) at an IRL of 7.0 cmH_2_O/L/s	4.6 ± 2.4	5.5 ± 2.9	4.4 ± 2.3	3.7 ± 1.9	0.219
IP (cmH_2_O) at an IRL of 15.0 cmH_2_O/L/s	7 ± 4.2	7.8 ± 3.9	7.1 ± 4.7	6 ± 3.6	0.581
IP (cmH_2_O) at an IRL of 25.0 cmH_2_O/L/s	10.2 ± 6.7	12 ± 5.9	10.2 ± 8.1	8 ± 4	0.368
IP (cmH_2_O) at an IRL of 46.7 cmH_2_O/L/s	11.9 ± 7.7	13.4 ± 6.8	12 ± 9.6	9.7 ± 4.4	0.531
IP (cmH_2_O) at an IRL of 67.0 cmH_2_O/L/s	13.5 ± 9.3	14.3 ± 8.7	13.9 ± 11.5	11.6 ± 5.6	0.773
IP (cmH_2_O) at an IRL of 78.0 cmH_2_O/L/s	14.6 ± 8.9	16.5 ± 10	14.1 ± 9.5	13.1 ± 6.2	0.637
IP (cmH_2_O) at an IRL of 0.6 cmH_2_O/L/s	4.5 ± 2.6	4.6 ± 2.1	5.1 ± 3.3	3.4 ± 1.1	0.255

BMI: body mass index; 6MWD: six-minute walk distance; 6MWT: six-minute walk test; IP: inspiratory pressure; and IRL: inspiratory resistive load.

a Values expressed as mean ± SD, except where otherwise indicated.

b Pearson's chi-square test for proportions; one-way ANOVA for quantitative variables; and Kruskal-Wallis H test for ordinal variables.

c Determined on the basis of the score on the International Physical Activity Questionnaire-long form.

Spearman's correlation coefficient between the inspiratory pressure and MIP generated at inspiratory resistive loads of 0.6, 7.0, 15.0, 25.0, 46.7, 67.0, 78.0, and 0.6 cmH_2_O/L/s was 0.04, 0.05, 0.08, 0.11, 0.12, 0.13, 0.15 and 0.05, respectively. Spearman's correlation between Borg scores and inspiratory pressure/MIP was not significant (p > 0.05).

## Discussion

The main finding of this cross-sectional study was that the scores for the perception of dyspnea induced by inspiratory resistive loads presented wide variability in healthy subjects. Among the 42 subjects who completed the test up to a load of 46.7 cmH_2_O/L/s, the perception of dyspnea was classified as low (or blunted) in 13 (31%), intermediate in 19 (45%), and high in 10 (24%). In addition, the level of perception of dyspnea was not found to be associated with age, gender, BMI, IPAQ-long form score, maximal respiratory pressures, or pulmonary function test results.

In the present study, dyspnea was successfully induced in healthy subjects through the application of inspiratory resistive loads of increasing magnitude, which significantly increased inspiratory pressure. These findings correspond with the reported typical effects of inspiratory resistive loads, which increase inspiratory effort and the overall work of breathing.^(^ 15 ^)^ We used a protocol with seven different inspiratory resistive loads, ranging from 0.6 to 78.0 cmH_2_O/L/s. The fact that we performed the test without pauses could explain why many subjects failed to complete all of its phases. When the inspiratory resistive load returned to 0.6 cmH_2_O/L/s at the end of the test, dyspnea scores decreased in all subjects but remained higher in the high perception group than in the other groups. This could be explained by the multidimensional aspects of dyspnea, as well as by the differences between the sensory and emotional aspects of its perception.^(^ 15 ^)^

It is worth noting that our approach differed from those taken in previous studies^(^ 13 ^,^ 14 ^,^ 16 ^,^ 24 ^)^ in that we did not apply a randomized sequence of inspiratory resistive loads. In the present study, we used inspiratory resistive loads of progressive magnitude in order to simulate the character of naturally occurring dyspnea. However, randomized presentations of different inspiratory resistive loads might be an alternative method that would avoid subject perception of the progressive magnitude of the loads.

Simon et al.^(^ 25 ^)^ investigated whether dyspnea induced in healthy subjects by different stimuli represents one or more than one sensation. The authors studied 30 subjects in whom dyspnea was induced by eight different stimuli. One of the stimuli used was breathing with an inspiratory resistive load. Subjects breathed for 2 min through a device used in inspiratory muscle training with an inspiratory resistive load of 260-280 cmH_2_O/L/s, at flow rates ranging from 0.3 L/s to 0.5 L/s. The mean intensity rating of dyspnea on the modified Borg scale associated with breathing against inspiratory resistance was 6.5 ± 2.5 points.

Kikuchi et al.^(^ 6 ^)^ examined whether dyspnea and chemosensitivity to hypoxia and hypercapnia were factors in fatal asthma. Those authors studied 22 asthma patients (11 who had had near-fatal asthma attacks and 11 who had not) and 16 healthy subjects, scoring the level of perception of dyspnea on the Borg scale during breathing against inspiratory resistance ranging from 0 cmH_2_O/L/s to 30.9 cmH_2_O/L/s. During breathing against a resistance of 20.0 cmH_2_O/L/s, the Borg scale scores of the healthy subjects ranged from 1 to 6.

Paulus et al.^(^ 26 ^)^ examined the hypothesis that elite athletes, in comparison with control subjects, show attenuated insular cortex activation during an aversive interoceptive challenge. Those authors studied 10 elite adventure racers and 11 healthy subjects. The subjects breathed through an inspiratory resistive load of 40 cmH_2_O/L/s. The authors asked the subjects to rate their experience, using a 10-cm visual analog scale. The mean perceived intensity of dyspnea among the healthy subjects, as rated on the visual analog scale, was 5.1 ± 0.9 points.

Ebihara et al.^(^ 7 ^)^ quantified the sensation of dyspnea during breathing through inspiratory resistive loads of 10, 20, and 30 cmH_2_O/L/s in 479 Japanese community-dwelling elderly people with normal lung function. Patients were divided into tertiles according to the perception of dyspnea, which was classified as low in 153 subjects, intermediate in 160, and high in 166. The authors found that, among community-dwelling elderly people, a blunted perception of dyspnea was associated with hospitalization, high medical costs, and all-cause mortality.

The present study has some limitations. First, the cross-sectional study design precluded the examination of temporal relationships between the perception of dyspnea and clinical outcomes. Second, our sample was small, and further investigations, involving larger cohorts, are therefore needed in order to elucidate the mechanisms related to a blunted perception of dyspnea in healthy subjects.

In the present study, the finding with the greatest clinical implications was that nearly one third of the subjects failed to discriminate the perception of dyspnea. The significance of this finding, in terms of its effect on clinical outcomes and the factors involved, remains unknown. Screening for the perception of dyspnea in asymptomatic and healthy subjects might be a means of identifying the need for more careful medical follow-up in order to avoid greater health care costs and higher mortality.

In conclusion, the scores for the perception of dyspnea induced by inspiratory resistive loads in healthy subjects presented wide variability. The level of perception of dyspnea was classified as low in 31% of the subjects, as intermediate in 45%, and as high in 24%. In addition, the level of perception of dyspnea was not found to be associated with the IPAQ-long form score or with the 6MWD.
